# Harnessing the angiogenic potential of adipose-derived stromal vascular fraction cells with perfusion cell seeding

**DOI:** 10.1186/s13287-025-04286-6

**Published:** 2025-05-01

**Authors:** Gregory Reid, Giulia Cerino, Ludovic Melly, Deborah Fusco, Chunyan Zhang, Oliver Reuthebuch, Giulia Milan, Anna Marsano

**Affiliations:** 1 Cardiac Surgery and Engineering Group, Department of Biomedicine, University of Basel and University Hospital of Basel, 4031 Basel, Switzerland; 2 Department of Cardiac Surgery, University Hospital of Basel, 4031 Basel, Switzerland; 3 Department of Plastic and Hand Surgery, University Hospital of Zürich, 8091 Zurich, Switzerland

**Keywords:** Bioreactor, SVF, MSC, Perfusion, Vascularization, Regenerative medicine, Tissue engineering

## Abstract

**Background:**

The rapid formation and long-term maintenance of functional vascular networks are crucial for the success of regenerative therapies. The stromal vascular fraction (SVF) from human adipose tissue is a readily available, heterogeneous cell source containing myeloid lineage cells, mesenchymal stromal cells, endothelial cells and their precursors, and pericytes, which are important for vascular support. Previous studies showed that seeding SVF cells under perfusion and pre-culturing them on three-dimensional (3D) collagen sponges enhances the vascular cell component in vitro while accelerating vascularization and improving human cell engraftment in vivo compared to static pre-culture. However, generating a perfusion-cultured SVF patch over a 5-day period is both costly and challenging for clinical translation. To overcome these limitations, this study explores a no-pre-culture strategy by comparing perfusion-based seeding with static cell loading on 3D sponges. The hypothesis is that perfusion-based seeding enhances in vivo cell engraftment and angiogenic potential by loading different SVF cell subpopulations onto 3D scaffolds during the seeding process.

**Methods:**

SVF-cells are seeded onto collagen scaffold using two approaches: a closed system perfusion bioreactor for 18 h or static loading onto the sponge surface. The in vitro cell distribution and baseline cytokine profiles were evaluated. Subsequently, human cell engraftment and differentiation were assessed in vivo using a nude rat subcutaneous implantation model. Analyses included the survival of transplanted human cells, the functionality and maturation of newly formed blood vessels within the SVF-patch.

**Results:**

Perfusion seeding significantly reduced the number of myeloid cells and achieved uniform spatial distribution across the construct. Vascular endothelial growth factor release was significantly increased following perfusion culture, whereas pro-inflammatory cytokines such as tumor necrosis factor-*α* and interleukin-1*β* were decreased. In the short term, perfusion culture enhanced uniform vascularization and SVF cell engraftment in vivo. However, the long-term differences between the perfusion-seeded and static-seeded groups diminished.

**Conclusion:**

Eliminating the need for prolonged pre-culture offers a feasible and cost-effective strategy for advancing regenerative cell-based therapies by reducing pre-culture times while preserving therapeutic efficacy. Perfusion-based seeding offers significant short-term benefits, including enhanced vascularization and cell engraftment, though long-term differences compared to static seeding are minimal. Further investigation is needed to evaluate its potential in a diseased ischemic heart model.

**Graphical abstract:**

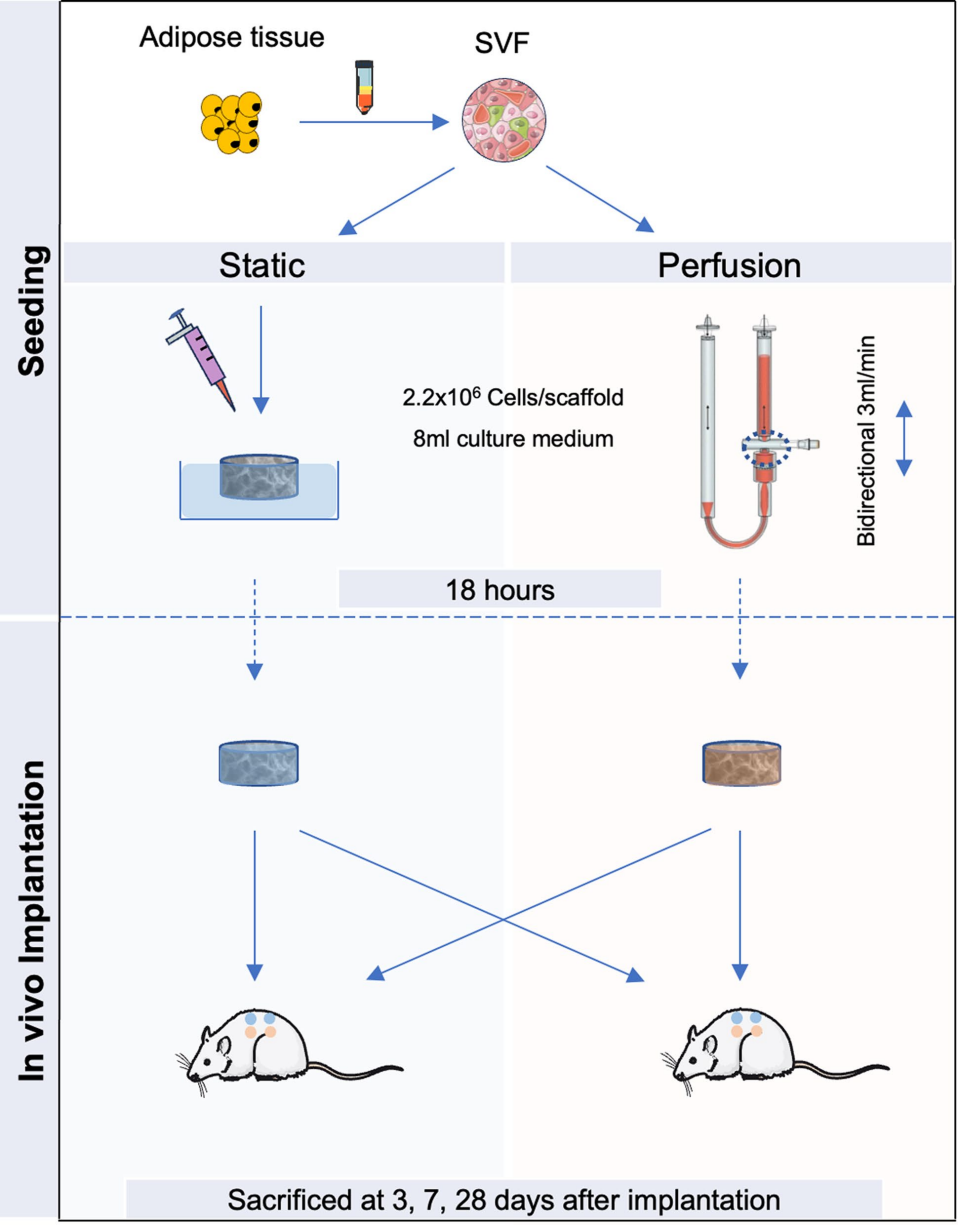

**Supplementary Information:**

The online version contains supplementary material available at 10.1186/s13287-025-04286-6.

## Background

The formation of a stable vascular network is a critical challenge in regenerative medicine strategies. Transplanted cells and tissues depend on the rapid self-assembly of vascular networks and their integration into the host vasculature, essential for ensuring long-term functionality and survival. A multicellular environment is required for the formation, recruitment, and sustained maintenance of blood vessels. Achieving a balance in the release of pro-inflammatory and anti-inflammatory cytokines is crucial for the local recruitment of host cells [1]. After an ischemic trauma, angiogenesis is crucial for blood flow recovery and ischemic tissue repair [2]

Stromal vascular fraction (SVF) is a readily available cell source derived from human adipose tissue. It is a heterogeneous cell mixture containing cells from the myeloid lineage, mesenchymal stromal cells [3], endothelial cells and their precursors, as well as pericytes. Due to its diverse cellular composition, SVF is considered a versatile tool in regenerative medicine, with the potential to contribute to tissue repair and regeneration. SVF has been used in various applications, including osteogenesis [4, 5], chondrogenesis [6], tenogenesis [7], and skin, wound healing [8, 9] and myocardial infarction repair [10]. These applications highlight the broad potential of SVF to promote tissue regeneration and enhance vascularization across a range of tissue types. Moreover, SVF has demonstrated an ability to modulate the inflammatory response in vivo [10–13]. The anti-inflammatory effect is particularly beneficial in regenerative therapies [14], as it fosters a more favorable environment for tissue repair [15]. In combination with other cell types, such as skeletal muscle cells, SVF has also been shown to enhance tissue formation and vascularization [16]. Additionally, SVF-derived nanovesicles have been found to increase neovascularization [17], which is crucial for the survival and integration of transplanted tissues [18].

Several recently developed strategies have been proposed to preserve and enhance the vasculogenic and angiogenic potential of SVF [19]. These include pre-vascularization, a process that involves the formation of vascular structures prior to transplantation [10]. SVF has been shown to facilitate the self-assembly of capillary structures, integrating into host integrated vascular networks [20, 21]. Another approach involves the selective enrichment of specific SVF cell subpopulations to enhance their regenerative potential [22, 23]. Furthermore, pre-culturing SVF under adverse conditions, such as hypoxia, has been demonstrated to boost its angiogenic capacity [24, 25]. Additionally, applying continuous flow of culture media through SVF-seeded porous scaffolds improves uniform cell distribution [26] while enhancing cell differentiation and tissue formation [27].

Our previous in vivo data revealed that SVF patches seeded under perfusion and subsequently pre-cultured for 5 days in a perfusion-based bioreactor exhibited superior cell survival and accelerated vascularization following subcutaneous implantation compared to static-pre-cultured SVF-based constructs [12]. These advantages have been attributed to the significant increase in CD45^−^CD34^−^CD146^+^ cell subpopulation within SVF-patches cultured under perfusion, underscoring the critical role of perfusion-culture phase in enhancing the angiogenic potential of SVF patches. However, the generation of perfused-SVF patches over a 5-day period poses logistical challenges and may limit their clinical translation [28]. To address this, our study aims to explore the differences and possible advantages of perfusion-based cell seeding versus static cell loading, by eliminating the 5-day pre-culture period and assess whether similar enhancements in vascularization can be achieved upon in vivo implantation of SVF patches. A direct comparison between perfusion- and static- seeding approach has not been previously investigated. This study seeks to determine whether these effects persist in vivo also with a static seeding technique without the need for a prolonged perfusion culture period. Importantly, transitioning to static seeding instead of perfusion seeding could offer significant practical advantages, including easier implementation in an operating room setting, reduced costs, and simplified handling logistics. Nevertheless, we hypothesize that perfusion-seeded patches will exhibit superior vascular potential due to the potential advantage of improved adhesion of SVF-derived vascular cell components under perfusion culture conditions compared to static seeding. To this end, we utilized a previously established nude rat subcutaneous model to investigate cell engraftment and the dynamics of vascularization [12, 13, 16, 29, 30]. This model serves as an ideal initial in vivo platform, providing a supportive vascular environment without the confounding factors often present in other in vivo models, such as the wound healing processes in ischemic models [31]. Additionally, the subcutaneous model enables the inclusion of four samples per rat, as opposed to a single sample, thereby minimizing the number of animals used while achieving the optimal goal of serving as a proof-of-concept to assess vascular potential. If validated, this no-preculture approach could facilitate clinical translation by greatly reducing pre-conditioning culture time and lowering the costs associated with generating a GMP-compliant manufacturing process for SVF patches.

## Methods

The work has been reported in line with the ARRIVE guidelines 2.0.

### SVF cell isolation

All materials were purchased from Sigma-Aldrich (Merck Millipore, Burlington, USA) unless otherwise stated. The lipoaspirate was digested with equal parts 0.15% w/v collagenase type II (Worthington Biochemical Corporation, Lakewood, USA) and phosphate buffered saline (PBS) (Invitrogen, Waltham, USA) at 37 °C undergoing continuous shaking for 60 min [11, 12]. Following centrifugation at 1,500 rpm for 10 min, the supernatant and the floating lipid-rich layer was discarded and the cellular pellet was washed once with PBS. The resulting cell suspension was strained through a 100 μm and a 70 μm nylon-mesh. The resulting SVF was then re-suspended in cell culture medium consisting of 87% v/v high glucose DMEM, 10% v/v fetal bovine serum, 1% v/v penicillin/streptomycin, 1% v/v glutamine, and 1% v/v N-2-hydroxyethylpiperazine-N-2-ethane sulfonic acid (HEPES) buffer. Nucleated cells stained with crystal violet were counted in an improved Neubauer chamber. Freshly isolated SVF cells were used straight away or frozen in 10% v/v dimethyl sulfoxide and 90% v/v FBS and stored in liquid nitrogen. To thaw, the cells were quickly heated in a 37 °C water bath and slowly resuspended in cell culture medium.

### Cell seeding and culture

Discs of 12 mm diameter and 3 mm thickness were cut from Ultrafoam® (BD, Franklin Lakes, USA) and hydrated for 24 h at 37 °C in culture medium. For both experimental groups, SVF cells were seeded at 2.2 million cells/scaffold suspended in 1 ml culture medium and cell culture was performed under standardized culture conditions at 37 °C, 97% humidity and 5% CO_2_.

For the static groups, the pre-hydrated Ultrafoam® discs were placed in the wells of a 6-well plate and cells were seeded on top of the discs using a pipette. After 30 min in incubator, 7 ml of culture medium was added to each well.

For the perfusion groups, the pre-hydrated Ultrafoam® discs were placed into a bioreactor (available from Cellec Biotek AG, Basel, Switzerland) [12] and held in place by two silicon O-rings (12 mm outer; 8 mm inner diameter), allowing for perfusion of the inner 8 mm of the Ultrafoam® discs. The cells were then introduced into the sealed bioreactor containing 7 ml of culture medium by means of the injection port and a bidirectional flow rate of 3 mL/min was started using a programmable syringe pump (PHD ULTRA 2000, Harvard Apparatus, Cambridge, USA). After 18 h culture time, the constructs were removed from the bioreactor aseptically [12]. A high seeding efficiency was achieved for all constructs, quantified by the counting the remaining cells in the cell culture medium.

### *In-vivo* animal experiments

Eight-week-old male nude rats (weight range 250 ± 16 g; Hsd: RH-rnu/rnu, Envigo) were anaesthetized by inhalation using a mixture of oxygen (0.3–0.6 L/min) and isoflurane (1.5–3 vol %). Four constructs, either static or perfusion based, were implanted in subcutaneous pockets in the dorsum of each rat (4 samples per experimental group and per time point). In each animal, two perfusion-seeded constructs and two static-seeded constructs were implanted symmetrically along the spine to minimize any potential confounding factors related to the animal or its location. A total of 10 rats were used (3 rats for the 3- and 7-time points and 4 rats for the 28-time point). Experimental groups include perfusion-seeded constructs and statically loaded patches as controls. No inclusion or exclusion criteria were adopted for this animal model. At the moment of sacrifice after 3, 7 and 28 days, rats were anaesthetized by intraperitoneal injection of a mixture of ketamine (1 mg/g) and xylazin (0.1 mg/g). Subsequently, the total rat vasculature was perfused with 1% w/v paraformaldehyde (PFA). In rats after 28 days following implantation, fluorescein isothiocyanate (FITC)-labeled Lycopersicon esculentum lectin 250 μg in 250 μl, (Vector Laboratories, Newark, USA) was injected into the femoral vein and allowed to circulate for 4 min prior to sacrifice by PFA [30]. The FITC-lectin labels all newly generated blood vessels connected to the main circulation with lectin, highlighting their functionality. Animal death occurred under anesthesia via exsanguination if lectin perfusion was performed. If rat perfusion was not carried out, rats were euthanized by CO_2_ inhalation. The constructs were harvested and fixed in PFA 4% w/v at 4 °C overnight and in sucrose 30% w/v at 4 °C for another day before embedding in optimal cutting temperature (OCT) compound (CellPath, Newtown, UK) and frozen in liquid nitrogen vapor. Animals were monitored for signs of pain, distress, and potential wound infections following predefined criteria (such as body weight, behaviour, posture, wound healing process). No signs of adverse effects or suffering were observed. Nevertheless, termination criteria were established in advance, in accordance with the license, to address any potential adverse events or signs of suffering. To further minimize potential distress in the animals, additional bedding was provided in the cages to prevent them from experiencing cold due to their lack of fur. The animals are kept in accordance with the FSVO regulations (Federal Food Safety and Veterinary Office). The rats are kept in hygienic, air-conditioned rooms that are equipped with modern cage systems, which provides to each cage with its own air supply to ensure hygiene entities.

### Analyses

No data was excluded.

#### Immunofluorescence

10 μm-thick sections were cut using a conventional cryostat (Leica Biosystems, Wetzlar, Germany) and stained according to standard protocols. In detail, slides were permeated with 0.3% Triton-X v/v solution for 10 min and then in blocking solution for 1 h containing either 2% v/v normal goat serum or 5% v/v donkey serum in PBS. All antibodies were diluted in blocking solution of 1% Bovine serum albumin (BSA). The following primary antibodies were added and left to incubate at 4 °C overnight: rabbit anti-Ki67 (Abcam, Cambridge, UK), mouse monoclonal anti-Human Nuclei (HuNu clone 235–1), rabbit anti-nerve/glial antigen 2 (NG2, Sigma-Aldrich), rabbit anti-cleaved caspase 3 (Cell Signaling technology, Inc., Danvers, USA), goat anti-vascular endothelial (Ve)-cadherin (Santa Cruz Biotechnology, Inc. Dallas, USA), rabbit anti-PECAM-1 (CD31, Abcam). All antibodies were diluted at 1:100, except anti-NG2 at 1:200. After washing, sections were then incubated at room temperature, covered from light for 1 h with 4’,6-diamidino-2-phenylindole (DAPI), Alexa488, Alexa546, or Alexa647 secondary antibodies (Invitrogen, Waltham, USA) at a dilution of 1:200. After washing, DAKO mounting medium (Agilent, Santa Clara, USA) was applied on the slide and the cover slip mounted. After drying at room temperature, slides were stored at − 20 °C. Fluorescence images were acquired at 20 × with Olympus BX63 microscope (Olympus, Shinjoku, Japan) or Zeiss LSM710 confocal microscope (Zeiss, Jena, Germany).

#### Image analysis

All image analyses were performed using Fiji 2.15 software (Open source, NIH, USA) or CellSens (Olympus) on at least triplicate samples for a total of 3 SVF donors and a range of 5–30 high-powered representative fields (acquired by a 20 × objective) per construct. Cell distribution and density was examined by the number and localization of DAPI positive cells relative to the total area of each field examined. In vivo primary read-outs were the vessel length density (VLD) and the human cell engraftment. The VLD was determined by normalizing the length of CD31 positive vessels to the total area of each field examined [12]. The human nuclei quantification was HuNu positive cells relative to the total area of each field examined. All other quantifications (cleaved caspase3, NG2-, Ki-67-, and Ve-Cadherin-positive cells) were deemed as a positive co-localization of the staining of interest with HuNu, relative to the total area of each field examined. Single-blind analyses were conducted during the in vitro and in vivo experiment.

#### Flow cytometry

The SVF cell phenotype was determined by 6-channel cytofluorimetric analysis before in-vitro cell culture [13]. Cells in suspension were chilled and incubated for 30 min with the following fluorochrome-conjugated antibodies to human markers: CD11b APC, CD31 FITC, CD34 APC Cy7, CD45 BV605, CD73 APC CD146 PE, CD90 FITC, and VEGFR2 PE (Biolegend, San Diego, USA) in staining buffer consisting of 0.5% v/v FBS and 2 mM EDTA in PBS. Per million cells, 5 μl were used for all the antibodies according to the manufacturer’s protocol. Data were acquired with LSR FortessaTM flow cytometer (BD Biosciences) and analyzed using Flowjo v10.1r5 software (FlowJo LLC, Ashland, USY). It was presented as percentage over the total number of live cells (DAPI positive) for both experimental groups.

#### In vitro assessment of cytokine release

Supernatants (7.7 ± 0.2 mL) were collected after 18 h hours of culture through the injection site for the perfusion condition or from the well for the static condition. Two samples were tested for each donor (*n* = 2) for all the experimental groups. The concentrations of released human Vascular endothelial growth factor (hVEGF), interleukin (IL)-6, IL-12, tumor necrosis factor (TNF)-*α* and IL-1*β* were measured using the respective ELISA kit (R&D Systems, Minneapolis, USA) according to manufacturer’s instructions. Data were expressed as picogram (pg) of protein normalized to the total amount of DNA (expressed in μg) for each relative construct.

#### Statistical analysis

Statistical analysis was performed using Prism 10 (Graphpad, Boston, USA) and statistical significance determined to be at * *p* < 0.05 and increased denominated with stars (** *p* < 0.01, *** *p* < 0.001, **** *p* < 0.0001). No data were excluded. All data were presented as standard errors of the mean (SEM) unless otherwise stated. Data sets were initially assessed for normal distribution using the Kolmogorov–Smirnov test. If found to be normally distributed, data was analyzed using a 2-tailed unpaired student's t test for single comparisons or analysis of variance one-way ANOVA for multiple comparisons, followed by Dunn’s or Tukey's post-hoc. Data not found to be normally distributed was analyzed using a non-parametric Mann–Whitney test for single comparisons or Kruskal–Wallis for multiple comparisons.

## Results

### In vitro analysis

#### Cellular distribution

**Fig. 1:**
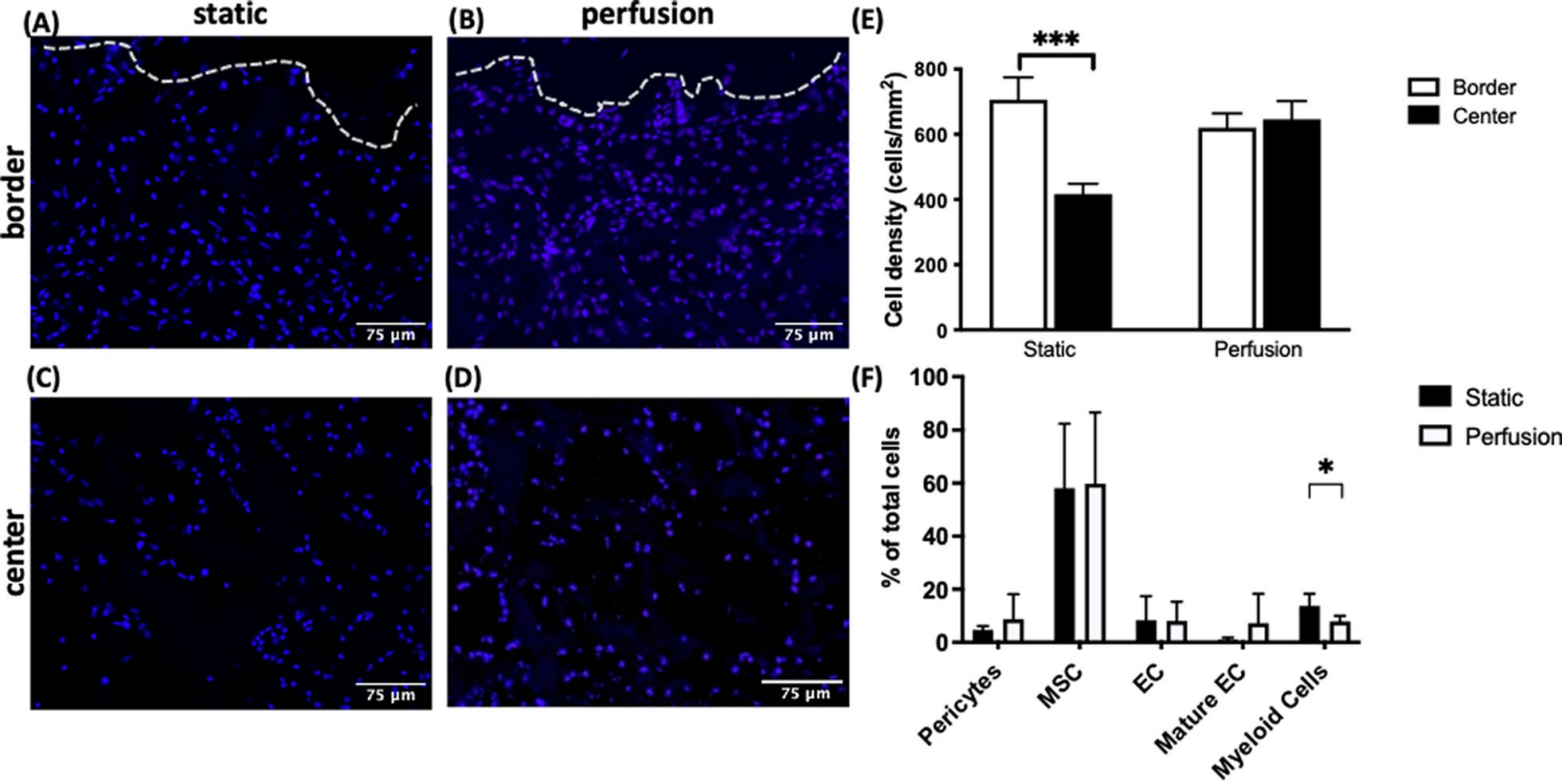
Cellular distribution and composition. **A–D** Representative images showing the cell distribution following cell seeding stained for DAPI in blue. Scale bar at 75 µm. **E** Cell density quantification at the border and center of the constructs, for static and perfusion conditions, respectively. Measurements were done following cell seeding for n = 4 samples from 2 independent experiments. **F** Relative cellular compositions of three SVF donors pooled were analyzed by flow cytometry of the SVF at day 0, after cell seeding yet before in vivo implantation for static and perfusion conditions respectively. Pericytes: CD45^−^, CD146^+^, CD34^−^; MSC: CD45^−^, CD73^+^, CD90^+^; progenitor EC (Endothelial cells): CD45^−^, CD34^+^, CD31^+^; Mature EC: CD45^−^, VEGFR2^+^, CD31^+^; Myeloid cells: CD45^+^, VEGFR2^+^, CD11b^+^. All statistical analyses were performed using an unpaired student's t-test. (* *p* < 0.05, *** *p* < 0.001)

At the end of the seeding process, cells were found to be equally distributed throughout the construct in the perfusion group, both at the border and the center of the construct. In the static group however, there was significantly lower cell density at the center (Fig. 1 A–D, E).

Further investigation of possible differences in relative cellular composition was performed by means of flow cytometry at day 0, after cell seeding yet prior to in vivo implantation (Fig. 1F). There was found to be a significantly higher percentage of myeloid cells (CD45^+^, VEGFR2^+^, CD11b^+^) in the static group, compared to the perfusion group. No difference was seen for the mesenchymal stromal cells (MSC, CD45^−^, CD73^+^, CD90^+^) or endothelial cells (EC, CD45^−^, CD34^+^, CD31^+^). On the other hand, in the perfusion groups, there was a trend towards higher percentages of pericytes (CD45^−^, CD146^+^, CD34^−^), and mature endothelial cells (mature EC, CD45^-^, VEGFR2^+^, CD31^+^), albeit without statistical significance. Representative plots and the exact values were listed in Figure S1 (Supporting Information).

#### Cellular profile

**Fig. 2:**
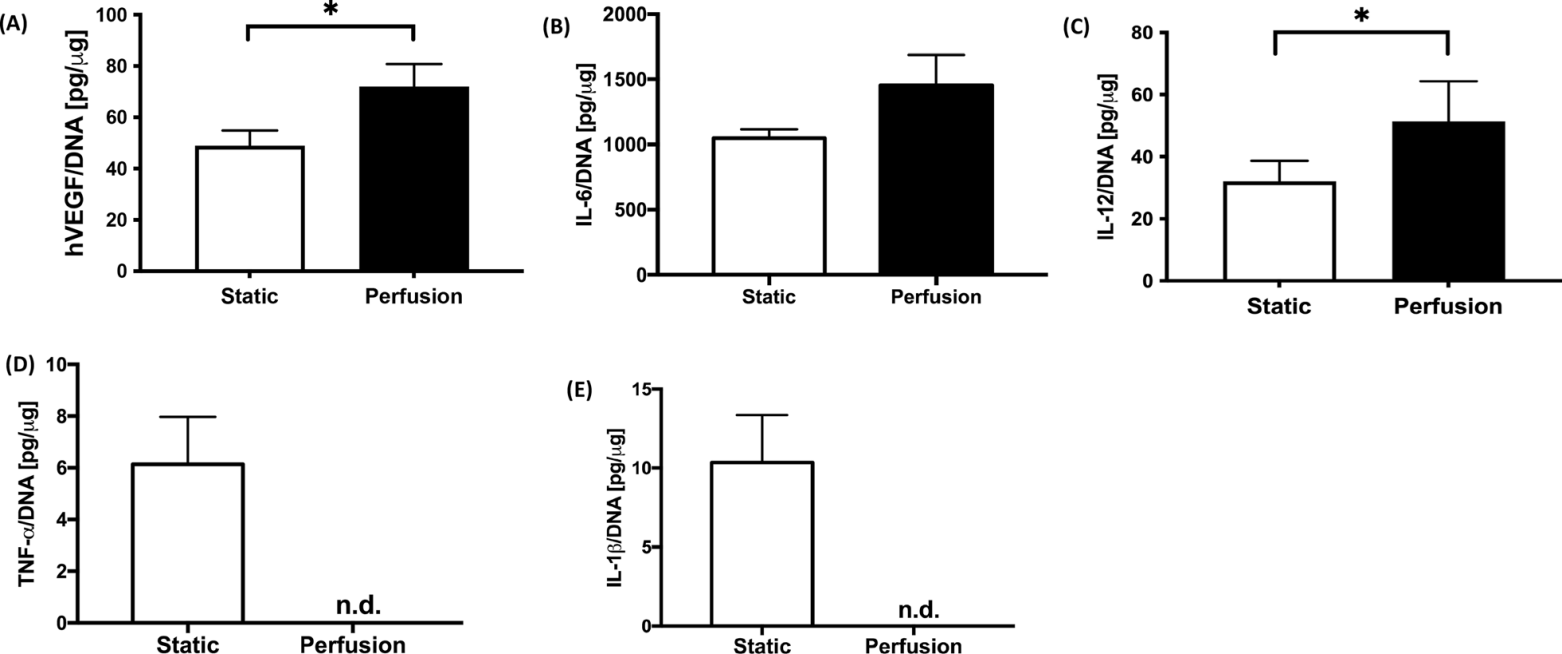
In vitro patch characterization: cytokine release. (**A**) Cytokine expression profile measured by ELISA in supernatants of static and perfusion conditions at time of implantation (day 0) of hVEGF (Static: 4 biological samples for 3 SVF donors; Perfusion: 3 biological samples for 2 SVF donors, and 1 biological sample for 1 SVF donor); (**B**) IL-6 (Static: 2 samples for 1 SVF donor, 4 samples for 2 SVF donors; Perfusion: 2 samples for 1 SVF donor, 3 samples for 1 SVF donor and 4 samples for 1 SVF donor); (**C**) IL-12 (Static: 4 samples for 2 SVF donors, 2 samples for 2 SVF donors; Perfusion: detected in 2 samples for 2 SVF donors; (**D**) TNF-α; (**E**) IL-1*β*. For both (D) and (E) Static: 3 samples for 1 SVF donor and 2 samples for 2 SVF donors resulted negative; Perfusion: 3 samples for 3 SVF donors not detected (n.d.). Statistical analysis was performed using a nonparametric Mann–Whitney test (* *p* < 0.05)

To establish any baseline differences between the two experimental groups, cytokine release profiles were established after cell seeding, by means of enzyme-linked immunosorbent assay (ELISA). Perfusion seeded cells, released significantly more hVEGF, than statically seeded cells (Fig. 2A). Analyzing further proinflammatory interleukins, both IL-6 (non-significant Fig. 2B) and IL-12 (*p* = 0.043, Fig. 2C) were released to a lesser degree by the static seeded cells. Typical pro-inflammatory markers TNF-*α* and IL-1*β* (Fig. 2D–E) were only expressed in the static group, values in the perfusion group being non detectable.

### In vivo analysis

#### Human cell engraftment

**Fig. 3:**
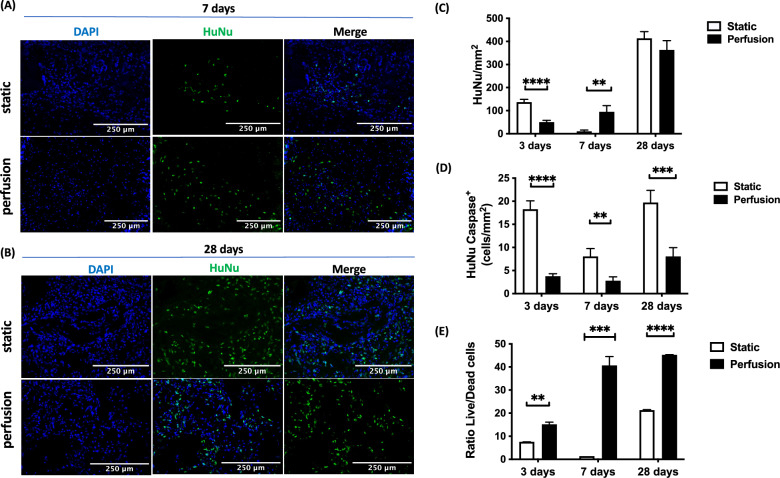
In vivo human cell engraftment. Representative immunofluorescent images stained for DAPI (blue) and HuNu (green) at 7 days (**A**) and 28 days (**B**). Scale bar at 250 µm. Quantitative analysis of HuNu^+^ cells (**C**). Quantitative analysis of HuNu and cleaved Caspase-3 co-expressing cells (**D**). Relative analysis of HuNu^+^ to cleaved Caspase3^+^ expressing cells (**E**). Statistical analysis was performed using a student's t-test (** *p* < 0.01, *** *p* < 0.001, **** *p* < 0.0001). Analyses were performed on n = 4 samples at 3 days, 4 samples at 7 days and 4 samples at 28 days

The presence and proliferation of human cells was determined, distinguishable from host rat cells by means of immunofluorescent human nuclei (HuNu) staining (Fig. 3A, B).

At day 3, in the static group a significantly higher overall number of human cells was present than in the perfusion group (Fig. 3C). This was true also at both the border and the center of the construct (Figure S2, Supporting Information), with a significant difference in between the two areas investigated. At day 7, the opposite could be seen, with a higher overall number of HuNu^+^ cells in the perfusion group. In the static group the overall number of cells decreased (Fig. 3C). This was also reflected in the quantification of apoptotic cells, deemed as the cells double positive for both HuNu and cleaved caspase 3. At all timepoints, there was significantly higher proportion of apoptotic cells in the static condition compared to the perfusion group (Fig. 3D). This also coincides with the calculated ratio of live to dead cells. For all time points, the perfusion group demonstrated a significantly higher ratio of live cells (Fig. 3E), equally distributed throughout the construct at the border and the center (Figure S2B; S2C, Supporting Information). At 28 days no difference was found for the overall number of cells in both groups (Fig. 3C), in contrast to the significantly greater number of apoptotic cells and lower ratio of living cells in the static group (Fig. 3D, E).

#### Human cell type characterization post implantation

**Fig. 4:**
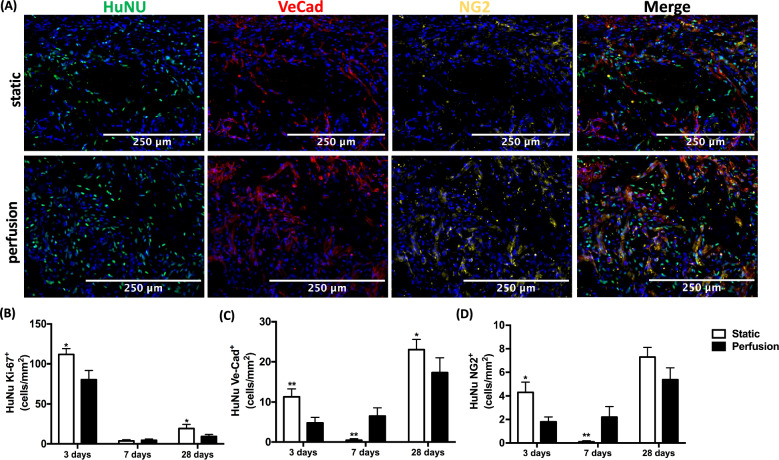
Human cell type characterization post implantation. (**A**) Representative immunofluorescent images at 28 days stained for DAPI (blue), HuNu (green), VeCad (red) and NG2 (yellow). Scale bar at 250 µm. (**B**) Quantitative analysis of HuNu^+^ and Ki67^+^ co-expressing cells. (**C**) Quantitative analysis of HuNu^+^ and Ve-Cad^+^ co-expressing cells. (**D**) Quantitative analysis of HuNu^+^ and NG2^+^ co-expressing cells. Statistical analysis was performed using a nonparametric Mann–Whitney test (* *p* < 0.05, ** *p* < 0.01). Analyses were performed on n = 4 samples at 3 days, 3 samples at 7 days and 6 samples at 28 days

Further immunofluorescent investigation of the individual cell types, positive for HuNu in the implanted construct was performed. Ki67, Ve-Cadherin and NG2 were used to identify proliferative cells, endothelial cells and pericytes respectively (Fig. 4A). At the 3-day timepoint, both experimental groups demonstrated a high number of Ki67 positive cells, statistically higher in the static group (Fig. 4B). This number declined greatly by day 7, with both groups showing very few Ki67^+^ cells at all. By 28 days there was again relevant cell proliferation, higher in the static group but manyfold lower than at day 3. When further analyzing any localization difference, only at day 3 day was there a significance between the border and center of the static constructs. This coincided with the previous HuNu quantification, being higher at the static border areas (Figure S3A, Supporting Information).

Further, Ve-Cadherin expression was found to be significantly higher at day 3 and day 28 in the static group (Fig. 4C), displaying again a near complete decline at day 7. Unlike the previous quantifications, there was a greater Ve-Cadherin expression at the center of the construct than at the border (Figure S3B, Supporting Information). In the perfusion group, expression levels remained relatively constant at the two early timepoints and did rise until day 28, yet lower than in the static group.

Overall NG2 levels displayed a very similar trend of Ve-Cadherin (Fig. 4D). At the early time point, expression levels were highest at the center of the static constructs (Figure S3 C, Supporting Information), only to nearly entirely decline at day 7. At the end time point, the static constructs displayed greater NG2 levels, though without significance.

#### In vivo patch vascularization

**Fig. 5:**
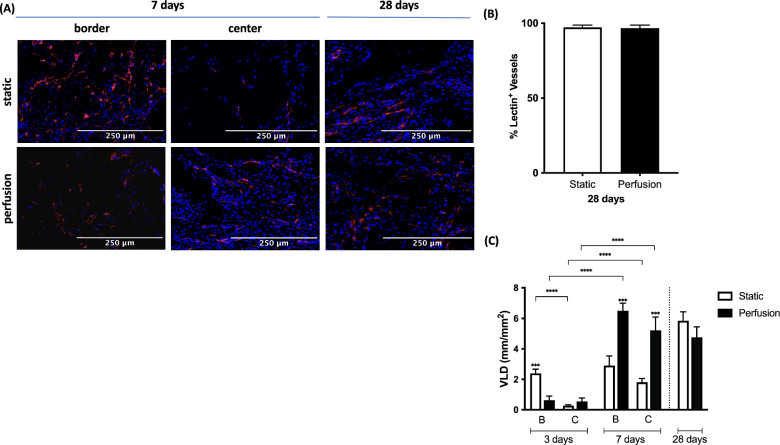
In vivo patch vascularization (**A**) Representative immunofluorescent images stained for DAPI (blue) and CD31 (red). Scale bar at 250 µm. (**B**) Quantitative analysis of lectin positive vessel-like structures at 28 days. (**C**) VLD analysis between the border and center of constructs. At 28 days no distinction is made for the border or center and analysis was performed on the whole construct. Statistical analysis was performed using a student's t-test and a one-way ANOVA (** *p* < 0.01, *** *p* < 0.001, **** *p* < 0.0001). Analysis for VLD was performed on n = 4 samples at 3 days, 3 samples at 7 days and 6 samples at 28 days. Analysis for % of lectin-positive vessels was performed on 5 samples at 28 days

In order to further investigate the functionality and maturity of the blood vessels formed in the construct, early and vascular endothelia cells were analyzed with a CD31 staining (Fig. 5A). From this, the vessel length density, VLD, was calculated. At the time of sacrifice on day 28, a fluorescent lectin (tomato) was intravenously injected, staining all vessels connected to the host vasculature. At 28 days, all vessels throughout the construct were positive for lectin, 97% ± 3.4 SD (93–100 range) and 97% + 4.8 SD (89–100 range) for the static and perfusion groups respectively (Fig. 5B).

The VLD area at 3 days was higher in the static group, yet lower at 7 days, both values being extremely significant (Fig. 5C). At 28 days however at 28 days, no difference was seen between the groups. Analysis of the different localizations, the borders and the center of the construct, were found to display a significant VLD area difference. In the static group, at the early time point of 3 days, the highest VLD was found at the borders, significantly higher than the center or any of the perfusion constructs analyzed. At 7 days however, like in the other analyses, the VLD area increased significantly in the perfusion group, both in the center and the border. The VLD area in the static condition remained constant at the borders, yet increased slightly in the center. The VLD in the perfusion border areas was twofold and at the center threefold greater than in the static group.

## Discussion

In this study, we demonstrated the enhanced vascular effect of hydrodynamic perfusion seeding on SVF in the mid-term after in vivo implantation. The microenvironment created by perfusion seeding led to lower implanted cell death and higher vascularization after 7 days, supporting our working hypothesis.

Interestingly, although statically seeded constructs had a significantly higher turnover and ratio of dead to live cells, the angiogenic effect evened out after 28 days in vivo. Full integration into the host vasculature was observed in both experimental groups. This suggests that while perfusion-based seeding provides a distinct early advantage, long-term vascular integration and cell survival are similar between static and perfusion-seeded constructs. Our findings suggest that perfusion seeding is particularly advantageous in situations requiring rapid vascularization, enhanced early cell integration, and uniform spatial distribution of both cells and vasculature. This includes cases involving the survival of vulnerable cell types (e.g. cardiomyocytes) or time-sensitive conditions, such as ischemic injuries or drug-induced tissue toxicity [32].

We previously demonstrated [12] a perfusion-induced selection and enrichment of the pericyte subpopulation after 5 days of continuous perfusion culture. In our results, we observed a trend towards an increase in the pericyte and mature EC population versus a significant reduction in the myeloid cell population. A selective enrichment of any cell type after such a short period seems unlikely. Shear stress imposed during perfusion culture is well known to activate the phosphorylation of endothelial p42/p44 MAPK/ERK [33]. These pathways could have been set in motion, explaining the stability and fast vessel formation observed in vivo (Fig. 5). The fact that no difference was observed in the long-term at 28 days could be due to the extensive remodeling of the implant. Indeed, no differences were evident between edges and center, and that at 28 days following the 5 days of culture, no differences between static and under perfusion culture [12]. The main difference between static and perfusion-based seeding process in cell composition was the washing and lower retention and adherence of CD45^+^ cells in the perfusion condition. This cell group is usually cultured in suspension conditions. Static seeding could therefore, lead to higher cell entrapment on the surface.

When analyzing the cytokine profile of both groups, the static culture exhibited a higher pro-inflammatory release seen in the TNF-α and IL-1*β* levels. Higher release profiles of VEGF and IL-6, both pro-angiogenic factors, might be explained by the stronger immune suppressive activity of the myeloid fraction present in the perfusion group [34–36]. This coincides with the previously mentioned release of acute phase pro-angiogenic factors, arguably by the myeloid cells present. Liao *et al.* recently also demonstrated the effect downstream of inhibition of pro-angiogenic factors such as from the VEGF group, in an in-vivo cardiovascular disease model [37].

When analyzing the presence of transplanted cells in vivo, at 3 days an overall higher number of HuNu^+^ cells was seen in static group. This was also the case for human Ve-Cad^+^ and NG2^+^ cells. A distinct reversal occurs in the mid-term at 7 days, with a very high cleaved caspase 3 expression demonstrating large scale cell death of the HuNu^+^ cells in the static group. A possible explanation could certainly be the pro-angiogenic environment selection mentioned previously. Therefore, a lack of viability dependent external growth factors and cytokines, also cell–cell interactions would activate apoptosis by means of the intrinsic pathway [38, 39].

The difference in VLD was highly significant at 7 days, indicating an organization of CD31^+^ cells. The selection bias of more mature ECs created by perfusion culture (Fig. 1), would certainly support this. A more ready integration into the host vasculature would support transplanted cells at an earlier time point. However, we also found that in the long-term after 28 days, any differences in absolute cell numbers, or individual cell populations or even VLD was evened out and equal in both groups. This again is proof of the powerful angiogenic capacity of SVF.

At 28 days following implantation, static and perfusion seeded constructs showed similar human cell presence and similar VLD. SVF-patches generated with a preculture under static conditions reached values of VLD and human cell engraftment of 5.9 and 414 cells per HPF respectively [12] This equals a 1.23-fold and 1.14-fold respective increase compared to constructs seeded under perfusion conditions. Patches generated under perfusion culture of 5 days also showed a high potential to accelerate ingrowth of blood vessels within the patch.

In a clinical setting, it is possible that a more rapid vascularization [40] and greater survival of transplanted cells could play a crucial role in the success of an implemented treatment, particularly in time-sensitive scenarios such as ischemic injury. This would equally be crucial in a complex setting, such as when a patient is on polypharmacy regime [41]. Our findings suggest that perfusion-seeded constructs demonstrate a significant vascular advantage in the mid-term at 7 days. This could suggest an improved early engraftment and patch vascularization. However, these differences are no longer observed at 28 days, suggesting that further study is needed to determine whether this early advantage translates into improved functional outcomes in a disease-relevant model. To further validate the therapeutic impact of perfusion-based seeding, it would be beneficial to create an in vivo model of clinically relevant tissue damage, such as myocardial ischemia. This could help determine whether the early vascularization advantage of perfusion-seeded constructs provides a meaningful regenerative benefit under pathological conditions. Additionally, employing a non-immunocompromised model with autologous cells could better reflect real-world translational applications.

## Conclusions

In conclusion, our study investigated the impact of perfusion seeding without preculture of SVF on the angiogenic potential, after subcutaneous in vivo implantation. Initial differences in cellular composition and reduced inflammatory and pro-angiogenic cytokine profiles favouring perfusion were found to favourable in the mid-term at 7 days, supporting our hypothesis that perfusion seeding improves short-term cell engraftment and vascularization. However, by 28 days, both static and perfusion-seeded constructs showed similar vascular density and human cell engraftment, indicating that the long-term benefits of perfusion-based seeding may not be sustained. While early advantages were observed, these did not translate into long-term measurable differences between the groups. Both static and perfusion seeding could be considered, with the former having the advantage of being a procedure that could be done in one day without the generation of the patch under GMP-conditions, but directly in the operating theatre. These findings underscore the potential of perfusion seeding in promoting rapid vascularization and cell engraftment in the mid-term, offering valuable insights for clinical applications requiring expedited tissue regeneration and immune modulation.

## Supplementary Information

Additional file 1.

## Data Availability

There are no sequencing datasets in this study. The data supporting the results reported in the paper are available from the corresponding author upon reasonable request. As such, there are no data archived in a public repository or included as supplementary files.

## Abbreviations

DMEM: Dulbecco’s modified eagle medium
EC: Endothelial cell
GMP: Good manufacturing practice
HPF: High powered field
hVEGF: Human vascular endothelial growth factor
HuNu: Human nuclei
HEPES: 4-(2-Hydroxyethyl)-1-piperazineethanesulfonic acid
MSC: Mesenchymal stromal cell
PBS: Phosphate buffered solution
PFA: Paraformaldehyde
SVF: Stromal vascular fraction
TNF-α: Tumor necrosis factor-alpha
VeCad: Vascular endothelial cadherin
VLD: Vessel length density
