# Supplementary material for: Harnessing the angiogenic potential of adipose-derived stromal vascular fraction cells with perfusion cell seeding
Source: Stem Cell Res Ther. 2025 May 1;16:220. doi: 10.1186/s13287-025-04286-6 (PMC12044990; doi:10.1186/s13287-025-04286-6)
Supplement: Supplementary file 1 — Additional file 1. [file 13287_2025_4286_MOESM1_ESM.docx]

**Supplementary Figures**


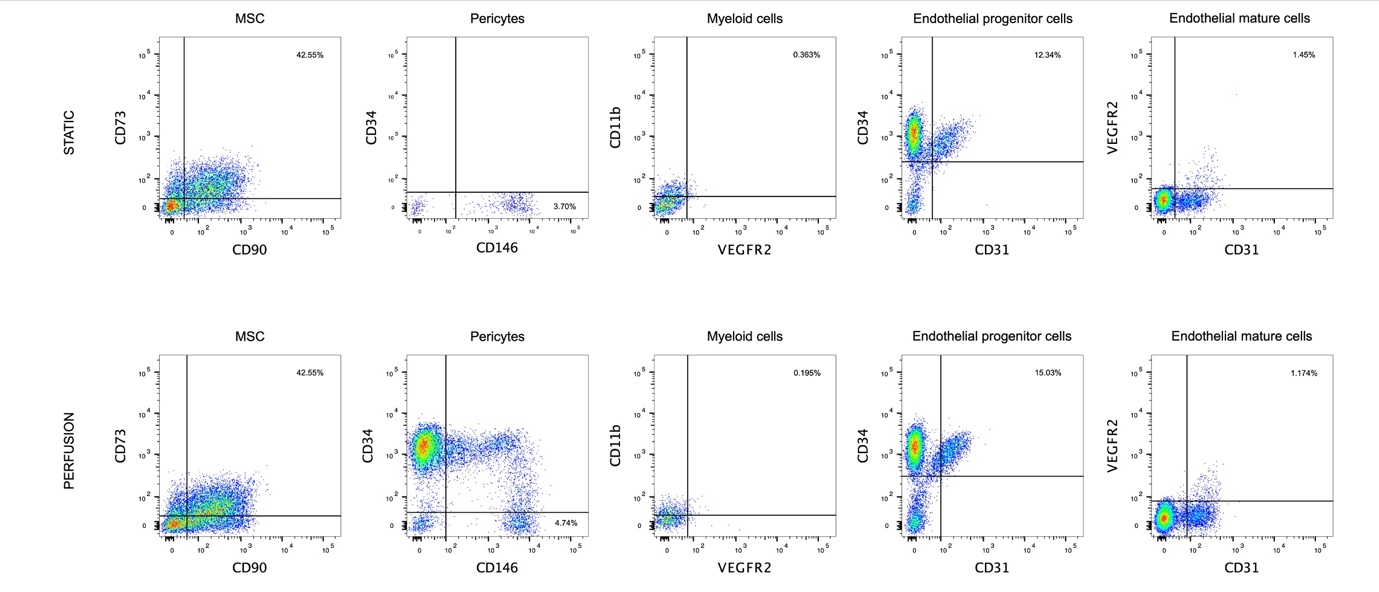


**Figure S1: Flow cytometry analysis of SVF donors**

Representative plots of the flow cytometry analysis of cellular composition, performed at day 0 before *in vivo* implantation. MSC: Mesenchymal stromal cells, EPC: Endothelial progenitor cells, Mature EC: Mature endothelial cells.


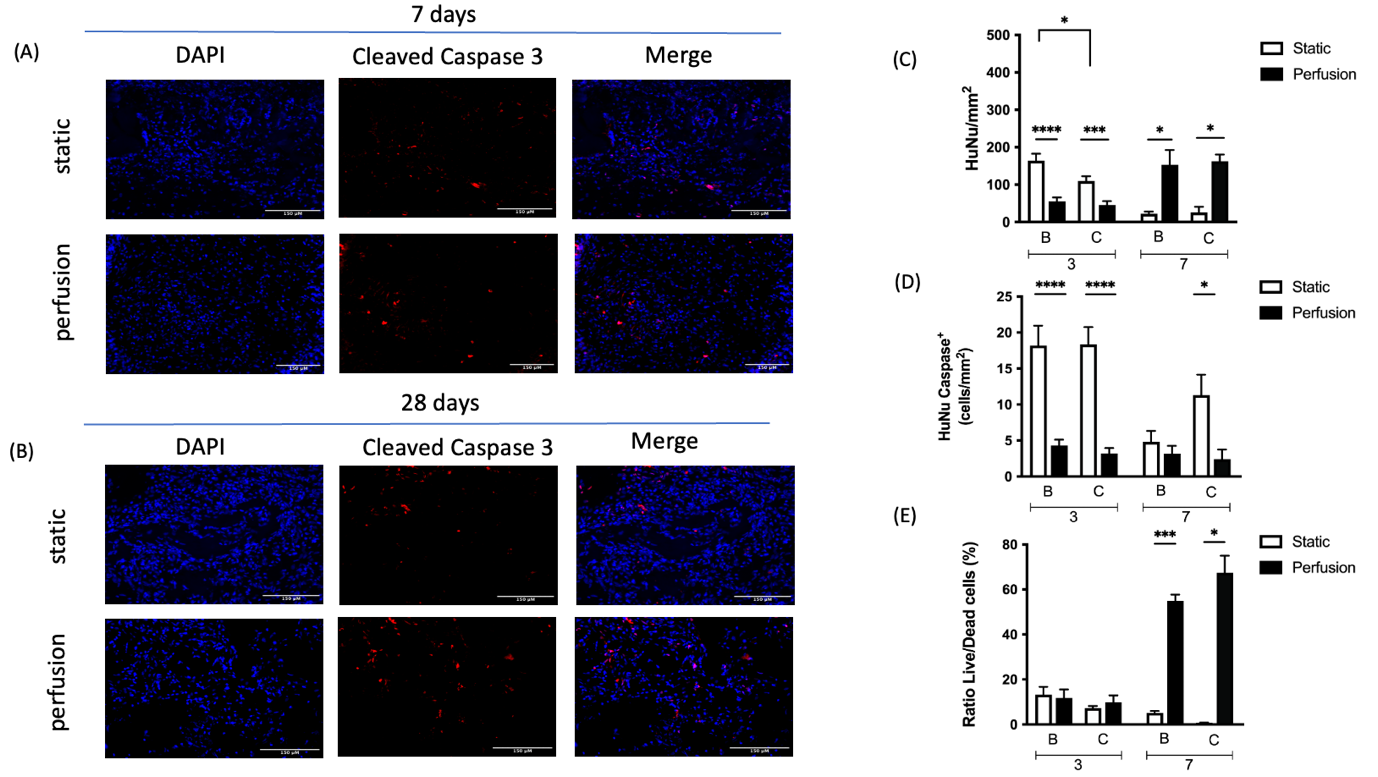


**Figure S2: *In vivo* human cell engraftment**

Representative immunofluorescent images stained for DAPI (blue) and cleaved-caspase-3 (red) at 7 days (**A**) and 28 days (**B**). Scale bar at 250 µm. Relative to construct border “B” and center “C” for static “S” and perfusion “P”: Quantitative analysis of HuNu^+^ cells (**C**). Quantitative analysis of HuNu and Caspase co-expressing cells (**D**). Quantification of HuNu^+^ to cleaved caspase-3^+^ expressing cells (E). Statistical analysis was performed using a student’s t-test (** *p* < 0.01, *** *p* < 0.001, **** *p* < 0.0001). Analyses were performed on days 3 and 7 for n = 4 and 3 samples respectively.


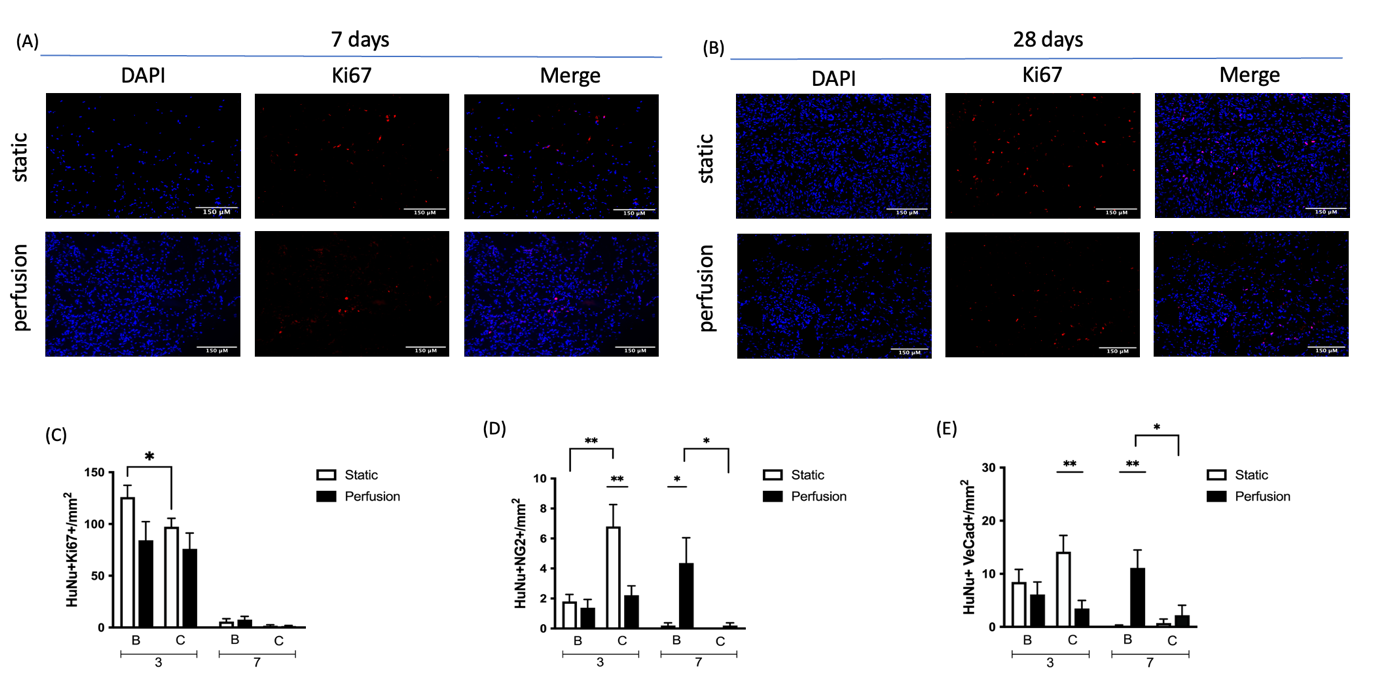


**Figure S3: Proliferation and human cell type characterization *in vivo***

Representative immunofluorescent images stained for DAPI (blue) and Ki67 (red) at 7 days (**A**) and 28 days (**B**). Scale bar at 250 µm. “S” indicates static, “P” indicates perfusion, “B” indicates Border and “C” indicates Center. Quantitative analysis of HuNu^+^/Ki67^+^ cells (**C**). Quantitative analysis of HuNu^+^/NG2^+^ co-expressing cells (**D**). Quantitative analysis of HuNu^+^/VeCad^+^ co-expressing cells (**E**). Statistical analysis was performed using a student’s t-test (* *p* < 0.05, ** *p* < 0.01). Analyses were performed on days 3 and 7 for n = 4 and 3, respectively.
